# Associations between Air Pollution Exposure and Blood Pressure during Pregnancy among PRINCESA Cohort Participants

**DOI:** 10.3390/toxics11050424

**Published:** 2023-05-03

**Authors:** Miatta A. Buxton, Safa Heydarzadeh, Carina J. Gronlund, Marisol Castillo-Castrejon, Myrna Souraye Godines-Enriquez, Marie S. O’Neill, Felipe Vadillo-Ortega

**Affiliations:** 1 Department of Epidemiology, School of Public Health, University of Michigan, Ann Arbor, MI 48109, USA; sheydarz@umich.edu (S.H.); gronlund@umich.edu (C.J.G.); marieo@umich.edu (M.S.O.); 2 Institute for Social Research, Survey Research Center, University of Michigan, Ann Arbor, MI 48104, USA; 3 Department of Pathology, Stephenson Cancer Center, Harold Hamm Diabetes Center, University of Oklahoma Health Sciences Center, Oklahoma City, OK 73104, USA; 4 Instituto Nacional de Perinatología Isidro Espinosa de los Reyes, Mexico City 11000, Mexico; dra.myrnagodines@gmail.com; 5 Department of Environmental Health Sciences, School of Public Health, University of Michigan, Ann Arbor, MI 48109, USA; felipe.vadillo@gmail.com; 6 Unidad de Vinculación Científica de la Facultad de Medicina, Universidad Nacional Autónoma de México en el Instituto Nacional de Medicina Genómica, Mexico City 14610, Mexico

**Keywords:** maternal health, ambient air pollution, trimester-specific exposure, blood pressure, cardiovascular health risks

## Abstract

High blood pressure (BP) is a risk factor for hypertensive disease during pregnancy. Exposure to multiple toxic air pollutants can affect BP in pregnancy but has been rarely studied. We evaluated trimester-specific associations between air pollution exposure and systolic (SBP) and diastolic BP (DBP). Ozone (O_3_), sulfur dioxide (SO_2_), carbon monoxide (CO), nitrogen dioxide (NO_2_), and particulate matter less than 10 and 2.5 μm in aerodynamic diameter (PM_10_, PM_2.5_) in the Pregnancy Research on Inflammation, Nutrition, & City Environment: Systematic Analyses (PRINCESA) study. Multipollutant generalized linear regression models with each pollutant and O_3_ were fit. Due to nonlinear pollution/BP associations, results are presented for “below the median” or “above the median”, where the beta estimate is the change in BP at a pollutant’s median versus BP at the pollutant’s minimum or maximum, respectively. Associations varied across trimesters and pollutants, and deleterious associations (higher blood pressure with higher pollution) were found only at pollutant values below the median: for SBP with NO_2_ in the second and third trimesters, and PM_2.5_ during the third trimester, and for DBP, PM_2.5_^,^ and NO_2_ in the second and third trimesters. Findings suggest that minimizing prenatal exposure to air pollution may reduce the risks of changes in BP.

## 1. Introduction

High blood pressure among normotensive individuals is a risk factor for hypertensive disorders of pregnancy, a condition that affects more than 8% of pregnancies in the United States [1]. In other parts of the world, such as Latin America, the prevalence and impact of high blood pressure on pregnancy health, including maternal mortality, is even greater [2]. Increased blood pressure is a key and common component in all hypertensive disorders of pregnancy, including chronic hypertension, gestational hypertension, and severe forms of pre-eclampsia and eclampsia [3], which are associated with maternal mortality [4] and morbidity as well as infant mortality [5]. In addition, hypertension during pregnancy is associated with other outcomes such as stroke [6,7] and chronic kidney disease [8] and has negative impacts on maternal health. For example, even though gestational hypertension, a form of hypertension that develops after the 20th week of pregnancy and generally resolves by the 12th week after pregnancy [9,10], is associated with problems beyond pregnancy and can affect long-term maternal health [7,11,12].

Although several changes that occur during pregnancy are physiological, risk factors such as obesity, age [13], and mental stress during pregnancy [14] have been linked to hypertension during pregnancy [15] and may lead to an exacerbation of pregnancy-related physiological changes. In addition, air pollution is an environmental hazard with toxic components that may contribute to increased risks of hypertensive disorders during pregnancy [16,17].

A limited body of research has linked exposure to air pollution with elevated blood pressure or hypertensive disorders of pregnancy [16,18]. Although previous studies on this subject have been performed, the effects of multiple pollutants have not been extensively examined. Many of the hypertensive disorders of pregnancy begin after mid-pregnancy [10,19], so examining how exposure to air pollution as early as the first trimester or even later during pregnancy is associated with high blood pressure may inform strategies to reduce maternal complications during pregnancy and improve maternal health in the long term. The aim of this study was to examine relationships between air pollution exposure during each trimester of pregnancy and blood pressure measured during each corresponding trimester. Specifically, we investigated associations between multiple ambient air pollutants and blood pressure in the following combinations: first trimester air pollution and first trimester SBP or DBP, second trimester air pollution and second trimester SBP or DBP, and third trimester air pollution and third trimester SBP or DBP.

## 2. Materials and Methods

### 2.1. Study Population

Data for this study are from the Pregnancy Research on Inflammation, Nutrition, and City Environment: Systematic Analyses (PRINCESA) cohort, which consists of 935 pregnant women from the Mexico City Metropolitan Area from 2009 to 2015. This metropolitan area has an estimated population of over 22 million and is located in a valley surrounded by mountains reaching elevations of 2240 m above sea level [20]. Women who participated in the PRINCESA cohort were 18 years or older and were recruited when they sought clinical care at public health clinics across the city and were invited to attend visits at a single hospital. An upper age limit was not set for this study. To be eligible for the study, women were asked to: provide the date their last menstrual period began, agree to prenatal visits every three to four weeks throughout their pregnancy, and provide written consent for their participation [20]. Women were excluded from enrolling in the study if they lived in a home outside of the coverage of the air-pollution monitoring network, had multiple fetuses, or had any medical or obstetric difficulties identified during the screening process, which occurred during the initial contact with the study team. Subsequently, if complications such as gestational diabetes or pre-eclampsia emerged among enrolled participants, women were referred for specialty care at the appropriate facilities. However, their data were recorded and used in applicable analyses. Additionally, women who were smokers were not eligible to enroll in the study, even though this criterion was later amended in the PRINCESA parent study to include 15 participants (1.6% of PRINCESA participants) who reported that they were active smokers at some point during pregnancy. Lastly, participants were included in the current study if they had a live birth. The PRINCESA study was approved by the University of Michigan Institutional Review Board and the ethics committees from the Secretaría de Salud del Gobierno de la Ciudad de México and the School of Medicine of the National Autonomous University of Mexico.

### 2.2. Data Collection

The initial visit for enrolled pregnant women included a screening questionnaire and informed consent. Follow-up occurred every three to four weeks for the remainder of their pregnancy [20]. At each visit, behavioral, informational, and anthropometric measurements, including blood pressure, were taken. Data on occupation, smoking, and secondhand smoke exposure were also collected.

### 2.3. Outcome

Blood pressure was measured and recorded during each visit. The study staff, who were appropriately trained to use a digital automatic blood pressure monitor, took blood pressure measurements. Participants were in a seated position when blood pressure was measured. Participants were asked to keep their feet flat on the floor and rest an arm on a table. The appropriate blood pressure cuff was selected to match the size of the participant’s arm. At each visit, blood pressure measurements were taken twice, approximately five minutes apart, and the average of two readings was recorded as the participant’s blood pressure in mmHg. We used blood pressure measures that were obtained at the latest visit that participants had in each trimester as the trimester-specific outcome.

### 2.4. Air Pollution Exposure Assessment

Air pollution data were obtained from the Mexico City Atmospheric Monitoring System or Sistema de Monitoreo Atmosférico de la Ciudad de México (SIMAT). SIMAT collects and makes available hourly data on six pollutants—ozone (O_3_, ppb), sulfur dioxide (SO_2_, ppb), carbon monoxide (CO, ppm), nitrogen dioxide (NO_2_, ppb), particulate matter less than 10 μm and 2.5 μm in aerodynamic diameter (PM_10_, PM_2.5_, μg/m^3^) from 34 stations [(http://ghdx.healthdata.org/record/mexico-mexico-city-automatic-air-quality-monitoring-network-database), accessed on 15 April 2015]. Although data were collected for most pollutants on an hourly basis, we utilized average daily concentrations (24 h concentrations) for PM_10_, PM_2.5_, SO_2_, and NO_2_ in our analysis. For CO and O_3_, the daily eight-hour running averages from all available monitoring stations were used. Air pollution exposure was estimated using Inverse Distance Weighting (IDW). The IDW method calculates anticipated concentrations at a given site as a weighted average of concentrations at nearby monitors [21]. At least five monitoring stations closest to the residence location were used to calculate the IDW exposure estimates, with a maximum of twelve for each pollutant. To calculate air pollution exposure for each trimester, daily air pollution estimates were used to calculate the mean of weekly pollutant values from the start of the corresponding trimester up to the point that each participant had the visit at which the blood pressure utilized for a given trimester was measured. The trimester-specific means air pollution value was then used to examine associations with trimester-specific SBP and DBP.

### 2.5. Covariates

Data were collected on other important obstetric and demographic factors among participants in the PRINCESA study. For the current study, we considered the following factors: gestational age at visit (months), maternal age at enrollment, marital status, education, exposure to secondhand smoke, number of previous pregnancies, and pre-pregnancy body mass index (BMI). We categorized maternal age at enrollment as 18–24 years, 25–34 years, and 35 years or older. Education was categorized as primary or no schooling, secondary and vocational/technical, or associate degree or higher. Marital status was characterized as single, which included single, widowed, and divorced individuals; married; and unmarried and living together. Gestational age at enrollment was categorized by trimester as first trimester (0–13 weeks), second trimester (14–26 weeks), and third trimester (27–40 weeks); all participants with gestational age data were enrolled either in the first or second trimester. The number of previous pregnancies was categorized into the following groups: ‘0′ indicating no previous pregnancies, 1–2 previous pregnancies, and 3 or more previous pregnancies. The following standard BMI categories were used for pre-pregnancy BMI for underweight (<18.5 kg/m^2^), normal weight (18.5−24.9 kg/m^2^), overweight (25.0−29.9 kg/m^2^), and obese (≥30.0 kg/m^2^).

### 2.6. Statistical Analysis

**Figure 1:**
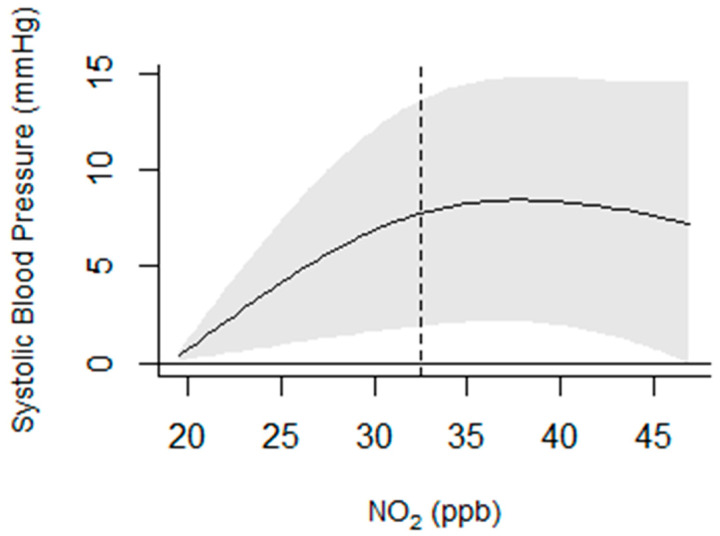
Sample Plot Showing Non-Linearity in the Relationship between NO_2_ and Systolic Blood Pressure: NO_2_ in the Second Trimester in a Model with O_3_. The dotted line represents the median.

We generated descriptive statistics to describe the demographic and obstetric characteristics of participants in the study. We examined the distribution of criteria pollutants for each trimester using the minimum, 25th, 50th, 75th percentiles, and maximum values and investigated correlations among pollutants using Pearson’s correlation. In addition, the mean and standard deviation were calculated for each pollutant. Similar distributions, in addition to the 5th and 95th percentiles, for SBP and DBP for each trimester were also generated. Finally, because initial diagnostics suggested that the association between pollutants and the BP outcomes was nonlinear (Figure 1), we used a spline with two degrees of freedom for the pollutant values in adjusted generalized linear regression models. We examined the relationships between first trimester air pollution and first trimester SBP and DBP; second trimester air pollution and second trimester SBP and DBP, and third trimester air pollution and third trimester SBP and DBP. We modeled change in blood pressure using the median level of air pollution as the cut-off point to create “below the median” and “above the median” categories. Therefore, our results are presented for “below the median”, where the beta estimate is interpreted as the change in blood pressure at the median level of a given air pollutant compared to blood pressure at the pollutant’s minimum level. In contrast, “above the median” is interpreted as a change in blood pressure at the maximum level of a given pollutant compared to blood pressure at the pollutant’s median level. Models were adjusted for the age of the mother at enrollment, exposure to secondhand smoke, pre-pregnancy BMI, marital status, and education. For second and third trimester models, we were not able to adjust for other periods of air pollution exposure due to multicollinearity signified by high variance inflation factors. False Discovery Rate (FDR) was used to adjust the *p*-values from the regression models to account for multiple testing. All statistical analyses were performed using SAS Statistical Software version 9.4 (SAS Institute Inc., Cary, NC, USA) and R version 3.3.3, (dlnm package, version 2.3.9).

## 3. Results

This study utilized data from 814 participants in the PRINCESA cohort who delivered a live infant. Table 1 shows the demographic and obstetric characteristics of participants. The majority of the participants were between the ages of 18 and 24 (53.6% of participants), while 44.4% and 33.5% of participants were normal weight and overweight, respectively, before the start of pregnancy.

Furthermore, most women were in the second trimester of pregnancy (14–26 weeks) at the time of enrollment in the study and accounted for 58.9% of participants. About half the participants had previously given birth to at least one child (49.9%), and 35.6% were in their first pregnancy. Although only 7.7% reported completing an associate degree or higher, 72.3% had secondary, technical, or vocational education. In terms of marital status, the majority of participants were living with their partners but were not married (52.8 %), while 25.1% were single, widowed, or divorced. Exposure to secondhand cigarette smoke was reported by 42.5% of participants, while 43% reported no exposure to secondhand smoke; data were missing for the remaining 14.5% of participants. The average number of visits for participants in this analysis was 2.25, and there were 199, 731, and 858 visits in the first, second, and third trimesters, respectively. A total of 11 participants were excluded from the analysis due to missing air pollution data.

Means, standard deviations, and select percentiles of individual pollutants are presented in Table 2. Pollutants were fairly stable across trimesters. PM_10_ was the only pollutant that exhibited more than a 10-point change in the values presented at any point during pregnancy. The maximum value for PM_10_ during the second trimester was 111 μg/m^3^ compared to 97 μg/m^3^ during the third trimester. Correlations among pollutants were >0.65, with O_3_ being the only exception (data not shown). Therefore, multipollutant models only included O_3_ as the second pollutant.

In addition, means, standard deviations, and select percentiles of the SBP and DBP for each trimester are shown in Table 3. Mean SBP and DBP were stable across trimesters, but differences in blood pressure were seen at both the minimum and maximum values. For SBP, the largest differences were seen between the first and second trimester minimum, 80 mmHg vs. 64 mmHg, and second and third trimester maximum, 122 mmHg vs. 160 mmHg, respectively. For DBP, the largest difference was seen for the second and third trimester maximum, 90 mmHg vs. 110 mmHg.

Associations between trimester-specific air pollution exposure and blood pressure varied across trimesters and pollutants. In multipollutant models, none of the pollutants were associated with SBP or DBP during the first trimester. However, statistically significant associations indicating harmful effects were found during the second and third trimesters for values below the median. Beta estimates and 95% confidence intervals reported for SBP (Table 4) and DBP (Table 5) are for pollutant 1. Deleterious effects on SBP were found below the median for NO_2_ (beta estimate (β) = 7.7) during the second trimester and (β = 11.8) during the third trimester. In addition, PM_2.5_ (β = 9.9) had a deleterious association with SBP during the third trimester below the median. For DBP, PM_2.5_ showed deleterious effects in the second (β = 8.8.) and third (β = 8.5) trimesters. Similar to PM_2.5_, NO_2_ was adversely associated with DBP in the second (β = 6.6) and third (β= 5.4) trimesters. Conversely, there was a consistent negative association between O_3_ both below and above the median, and SBP during the second and third trimesters.

## 4. Discussion

In this study of pregnant women from the PRINCESA study, we observed associations between trimester-specific ambient air pollutants and trimester-specific blood pressure. However, the associations differed across trimester and pollutants and were not consistently positive. Results were null for all pollutants during the first trimester. For SBP, the strongest deleterious association was found during the third trimester for values below the mean for NO_2_, thereby indicating the harmful effects of NO_2_ when SBP is compared at a median compared to the lowest value. However, the lack of deleterious associations above the median for all pollutants was contrary to our expectation that increased levels of pollution would be adversely and significantly associated with blood pressure. Air quality notification systems might help explain these unexpected findings. In Mexico City, alerts are issued to warn the public and particularly vulnerable groups when the Air Quality Index (AQI) reaches 150. Response to such public health alerts and/or potentially other visual evidence of adverse air, such as smog, ref. [22] might lead to increased adherence to preventive efforts activated by public health messaging.

Statistically significant negative associations were found for O_3_ both below and above the median, and these results were unexpected. To date, findings from previous studies have been inconsistent. Unlike our study, a study conducted in a cohort of 1684 pregnant women in Allegheny County, Pennsylvania, found that exposure to O_3_ during the first trimester was positively associated with increases in average SBP and DBP later in pregnancy among non-smokers [23]. In this study, CO, SO_2_, NO_2_, PM_10_, and PM_2.5_ were also examined, but associations were null for PM_2.5_, CO, SO_2_, and NO_2_. PM_10_ was associated with a change in SBP, but not DBP. In another study, O_3_ was positively associated with hypertensive disorders of pregnancy, a composite outcome that included gestational hypertension, pre-eclampsia, and eclampsia, all of which have hypertension as the common component across conditions [24]. However, a study conducted in Guangzhou, China among 4200 participants in a birth cohort found both positive and negative associations between early pregnancy exposure to O_3_ and blood pressure and hypertensive disorders of pregnancy [25]. Positive associations were found for the ninth month of pregnancy and hypertensive disorders of pregnancy, and statistically significant negative associations were found between O_3_ and hypertensive disorders of pregnancy during the second trimester [25]. Associations between O_3_ and DBP were positive during the first and second trimester, but only significant during the second trimester. Additionally, in a prospective cohort study conducted in the Netherlands among 7006 women, associations between exposure to air pollution and blood pressure during pregnancy also varied by pollutant and across trimesters. In this study, PM_10_ was not associated with SBP in the first trimester but was associated with increases in SBP in the second and third trimesters. However, PM_10_ was not associated with DBP at any point during pregnancy. On the other hand, NO_2_ was associated with SPB during all trimesters of pregnancy but not with DBP [26]. In yet another study conducted among 817 pregnant women in Ghana, West Africa, CO was positively associated with DBP but not SBP [27].

Air pollutants may cause damage to the cardiovascular system through acute effects such as changes in the autonomic nervous system, activation of pulmonary and systemic inflammation, and oxidative damage to vascular cells or through long-lasting mechanisms such as endothelial dysfunction and increased blood coagulability [28,29]. Blood pressure during pregnancy is influenced by adaptations of the systemic maternal circulation to maintain the homeostasis of uteroplacental perfusion. These adaptations may exacerbate the effects of air pollution on cardiovascular health among pregnant women. In previous analyses conducted among participants in this cohort, we reported increases in pro-inflammatory serum cytokines associated with an increase in PM_10_ [30]. In addition, some of these effects can damage the placenta, especially during the first trimester of pregnancy, when placentation occurs [31,32]. This combination of effects may contribute to the pathogenesis of gestational hypertension, pre-eclampsia, and eclampsia. Additionally, hypothesized longer lasting mechanisms involving endothelial dysfunction may partly explain associations seen later in pregnancy during the second and third trimesters in our study. A majority of participants (53.6%) in our study were in the 18–24 age group; therefore, we had a larger proportion of individuals with a lower risk of increasing blood pressure because blood pressure increases with age [33]. Unfortunately, we were unable to evaluate associations by age group (or even by BMI categories) due to the small sample sizes in each age group per trimester.

Our findings have implications for maternal and child health outcomes and even maternal health in the long term. Previous studies have shown that high blood pressure and blood pressure-related conditions that occur during pregnancy are associated with multiple adverse maternal and child health outcomes [7,34,35]. Complications from hypertension during pregnancy can lead to preterm delivery [36,37], which may lead to a range of health problems during infancy and beyond, particularly among infants born earlier in pregnancy [38,39,40,41]. Options for treating hypertension during pregnancy include antihypertensive medications that are dually aimed at reducing blood pressure to protect maternal health and limiting potential side effects for the fetus [42,43]. In extreme cases such as pre-eclampsia and in instances when antihypertensive agents are not effective [44], delivery of the infant is indicated to protect the health of both mother and child [45]. Adverse outcomes coupled with limitations associated with treatment are key reasons focusing on prevention strategies may be an important avenue to explore to reduce the burden of hypertension during pregnancy. Numerous reports have documented improvements in human health after national and local reductions in air pollution [46]. For example, a number of these reports have focused on exploring a reduction in exposure by focusing on efficient filtration systems [47,48] or expanding current air pollution monitoring systems to include portable/mobile monitors to fill the limitations of current exposure assessment methods. The objective is that the collection of local-scale high-quality data may be useful for policy development [49,50]. Other reports of recent COVID-19-related increases in the prevalence of people working from home may lead to changes in post-COVID-19 commuting patterns [51] and offer potential opportunities to reduce air pollution. Considerations should be given to applying applicable efforts in maternal and child health improvement programs, particularly in communities with high rates of adverse maternal and child health outcomes.

Although many studies have evaluated associations between air pollution exposure and hypertensive disorders of pregnancy (combined or focused on one of the more severe conditions), not many studies focus on blood pressure as an outcome. Our study focuses on air pollution exposure and blood pressure during pregnancy and adds to the literature.

Our examination of associations between air pollution and blood pressure from each trimester, unlike previous research, which used average blood pressure from at least two visits [23,25], is a strength of this study. Another strength of this study is the statistical approach utilized in modeling air pollution; the relationship between air pollution and blood pressure was not linear, and this was used to inform how a pollutant was modeled. Additionally, the *p*-values reported in this study were adjusted to account for multiple testing. Lastly, the use of multipollutant models allowed for opportunities to model air pollution as it is experienced, even though we were limited to including only two pollutants per model due to concerns about collinearity. Potential limitations in our study should be considered. Data on blood pressure medication intake were not adjusted for because a small number of participants (*n* = 8) who were prescribed antihypertensive medications started medications later in pregnancy, which occurred after the visit dates utilized in this study. Additionally, even though we adjusted for education, we were unable to account for other important covariates, such as income, and because these variables were not available for PRINCESA participants. Furthermore, we did not control for personal smoking because the PRINCESA cohort included 1.6% (15/935) of women who reported smoking. Current smoker status was an exclusion criterion, but the study was later amended to include 15 participants who reported smoking at some point during pregnancy. For other potential covariates that were available such as temperature, due to the large number of terms included in the models, additional variables were not included if they did not meet the requirement for confounding or if their inclusion was not expected to change the results. Another limitation is that we were not able to adjust for other periods of air pollution during a given trimester because the variance inflation factors for terms in multi-trimester models, including seasonal control, were very high. This issue of multicollinearity precluded us from being about to account for the effects of the first trimester as distinct from those in the second trimester or third trimester during later pregnancy. Additionally, 14.5% of participants did not report information on secondhand smoking or the number of previous pregnancies, thus limiting the analytic sample size and resulting in a potential loss of statistical power. Finally, weight gained during pregnancy may affect blood pressure and the precision of the estimates produced in our analysis. We had a large number of terms in each model and decided to include variables that met the requirements of a confounder; since weight gained during pregnancy was not associated with air pollution, it was not included in the final models.

## 5. Conclusions

Exposure to air pollution was significantly associated with deleterious effects on SBP and DBP. Although associations varied across trimesters and by pollutants, and harmful associations were found only at pollutant values below the median for both SBP and DBP, these findings suggest that minimizing prenatal exposure to air pollution may reduce risks of changes in blood pressure. This research provides additional support for air pollution’s role in high blood pressure during pregnancy, which serves as the basis for severe forms of hypertensive disorders during pregnancy. A key component of controlling blood pressure during pregnancy is to focus on the prescription of antihypertensive medications, which have side effects [42], but minimizing maternal and prenatal exposure, particularly community-based approaches that lead to reduced exposure early in pregnancy, may serve as a complementary avenue to explore to decrease the risk of adverse outcomes.

## Figures and Tables

**Table 1:** Maternal Characteristics of Participants, PRINCESA Cohort 2009–2015.

Maternal Characteristics	N (%)
Age at enrollment (years)	
18–24	436 (53.6)
25–34	304 (37.4)
≥35	74 (9.1)
Pre-pregnancy BMI (kg/m^2^)	
Underweight (<18.5)	37 (4.6)
Normal Weight (18.5–24.9)	361 (44.4)
Overweight (25–29.9)	273 (33.5)
Obese (≥30)	142 (17.4)
Missing	1 (0.1)
Gestational age at enrollment (weeks)	
First trimester (0–13)	316 (38.8)
Second Trimester (14–26)	479 (58.9)
Third Trimester (27–40)	0 (0)
Missing	19 (2.3)
Education	
Primary or no schooling	83 (10.2)
Secondary/Vocational/Technical	588 (72.3)
Associate or higher	63 (7.7)
Missing	80 (9.8)
Marital Status	
Single/Widowed/Divorced	204 (25.1)
Married	177 (21.7)
Living together but not married	430 (52.8)
Missing	3 (0.4)
Number of Previous Pregnancies	
0	290 (35.6)
1–2	358 (44.0)
≥3	4 (5.9)
Missing	118 (14.5)
Secondhand smoke exposure (at home)	
Yes	346 (42.5)
No	350 (43.0)
Missing	118 (14.5)

**Table 2:** Distribution of Trimester-specific Air Pollution, PRINCESA Cohort 2009–2015.

Pollutant/Trimester	Mean (SD)	Min	25th	50th	75th	Max
PM_2.5_ (μg/m^3^)						
1st	25 (6)	13	20	26	29	38
2nd	24 (6)	12	20	24	29	42
3rd	24 (6)	11	19	24	29	44
PM_10_ (μg/m^3^)						
1st	57 (16)	29	44	57	67	105
2nd	55 (16)	26	41	54	66	111
3rd	53 (16)	23	39	52	66	97
NO_2_ (ppb)						
1st	34 (5)	22	29	34	38	46
2nd	32 (5)	19	28	32	36	47
3rd	32 (6)	18	27	31	36	52
SO_2_ (ppb)						
1st	5.9 (1.8)	2.4	4.6	5.8	7.1	10.3
2nd	5.5 (1.8)	2.3	4.1	5.2	6.6	11.4
3rd	5.6 (2.2)	2.0	3.9	5.1	7.1	19.3
O_3_ (ppb)						
1st	57 (10)	39	49	54	65	81
2nd	57 (10)	39	49	54	64	90
3rd	55 (10)	33	48	52	57	89
CO (ppm)						
1st	1.6 (0.3)	0.8	1.3	1.6	1.8	2.6
2nd	1.5 (0.3)	0.9	1.2	1.4	1.7	2.4
3rd	1.5 (0.3)	0.8	1.2	1.4	1.7	2.6

**Table 3:** Distribution of Trimester-specific Systolic and Diastolic Blood Pressure Among Participants in the PRINCESA Cohort, 2009–2015.

BP Measure/Trimester	Mean (SD)	Min	5th	25th	50th	75th	95th	Max
Systolic Blood Pressure								
1st	99 (10)	80	85	90	100	110	118	132
2nd	99 (10)	64	85	90	100	108	110	122
3rd	101 (10)	66	85	90	100	110	120	160
Diastolic Blood Pressure								
1st	65 (8)	48	55	60	60	70	80	100
2nd	63 (7)	38	52	60	60	70	80	90
3rd	66 (8)	40	56	60	64	70	80	110

**Table 4:** Association between Trimester-specific Air Pollution Exposure and Trimester-specific Systolic Blood Pressure (mmHg) Among Participants in the PRINCESA Cohort, 2009–2015.

	1st Trimester		2nd Trimester		3rd Trimester	
Pollutant 1	Below Median	Above Median	Below Median	Above Median	Below Median	Above Median
PM_2.5_	7.2 (−4.9, 19.4)	6.9 (−2.5, 16.3)	2.2 (−4.8, 9.2)	−4.6 (−12.3, 3.0)	9.9 (2.9, 16.9) *	−6.1 (−15.2, 3.0)
PM_10_	−7.3 (−17.4, 2.8)	7.1 (−1.4, 15.6)	−3.2 (−8.7, 2.3)	−8.6 (−15.5, −1.6)	−3. (−9.8, 3.5)	−5.4 (−12.0, 1.2)
NO_2_	11.2 (0.1, 22.2)	−1.1 (−10.9, 8.6)	7.7 (1.9, 13.6) *	−0.5 (−6.6, 5.5)	11.8 (5.9, 17.6) ***	−3.6 (−12.3, 5.1)
SO_2_	0.3 (−9.9, 10.6)	3.2 (−4.8, 11.3)	−0.1 (−4.8, 4.6)	2.3 (−4.3, 8.9)	−1.1 (−5.4, 3.2)	2.8 (−13.1, 18.6)
CO	−10.8 (−23.8, 2.1)	7.0 (−3.7, 17.6)	−8.0 (−12.4, −3.7) ***	−3.4 (−9.7, 2.8)	−3.1 (−8.2, 2.0)	−3.0 (−11.0, 4.9)
O_3_	−2.2 (−8.7, 4.3)	−1.6 (−10.8, 7.6)	−7.7 (−11.5, −3.8) ***	−20.8 (−27.8, −13.8) ***	−10.4 (−15.7, −5.1) ***	−12.1 (−18.9, −5.2) **
O_3_	−1.7 (−8.3, 4.9)	−2.0 (−11.2, 7.2)	−5.7 (−9.5, −1.8) *	−19.9 (−26.8, −13.0) ***	−9.3 (−14.7, −4.0) **	−12.2 (−19.3, −5.2) **
O_3_	0.7 (−6.5, 7.8)	−1.3 (−11.1, 8.5)	−6.0 (−10.2, −1.8) *	−17.1 (−24.7, −9.6) ***	−8.1 (−14.1, −2.0) *	−10.0 (−17.6, −2.4) *
O_3_	−4.0 (−11.3, 3.4)	−8.1 (−18.8, 2.5)	−7.4 (−11.5, −3.4) ***	−19.2 (−27.8, −10.5) ***	−13.5 (−19.4, −7.6) ***	−12.1 (−20.5, −3.8) *
O_3_	−1.7 (−8.2, 4.9)	−2.1 (−11.4, 7.3)	−7.1 (−11.1, −3.2) ***	−21.7 (−28.7, −14.6) ***	−9.3 (−15.0, −3.7) **	−13.6 (−20.7, −6.5) ***

Beta estimates and 95% confidence intervals (β [95% CI]) are for pollutant 1 from models that included a second pollutant . Multipollutant models were adjusted for the age of the mother at enrollment, exposure to secondhand smoke, pre-pregnancy BMI, marital status, and education. Bold font indicates statistical significance at * *p* < 0.05, ** *p* < 0.01, *** *p* < 0.001. *p*-values were adjusted for multiple testing using the False Discovery Rate.

**Table 5:** Association between Trimester-specific Air Pollution Exposure and Trimester-Specific Diastolic Blood Pressure (mmHg) among Participants in the PRINCESA Cohort, 2009–2015.

	1st Trimester		2nd Trimester		3rd Trimester	
Pollutant 1	Below Median	Above Median	Below Median	Above Median	Below Median	Above Median
PM_2.5_	5.7 (−1.2, 12.6)	3.0 (−4.2, 10.2)	8.8 (4.8, 12.9) ***	−3.6 (−9.4, 2.2)	8.5 (3.8, 13.2) ***	0.7 (−6.8, 8.2)
PM_10_	2.4 (−3.8, 8.5)	2.7 (−4.0, 9.3)	1.6 (−2.0, 5.2)	−4.4 (−9.8, 0.9)	3.0 (−1.5, 7.6)	2.7 (−2.7, 8.1)
NO_2_	5.3 (−0.6, 11.2)	1.5 (−5.9, 8.9)	6.6 (3.0, 10.2) ***	−2.6 (−7.3, 2.0)	5.4 (1.5, 9.4) *	−1.1 (−8.3, 6.2)
SO_2_	3.0 (−2.8, 8.7)	6.6 (0.5, 12.7)	3.1 (0.4, 5.8)	3.2 (−1.9, 8.2)	1.9 (−0.9, 4.7)	12.4 (−0.7, 25.6)
CO	3.2 (−4.2, 10.6)	5.2 (−2.9, 13.2)	−0.2 (−2.9, 2.5)	−1.3 (−6.2, 3.5)	−0.1 (−3.6, 3.4)	3.3 (−3.2, 9.8)
O_3_	−3.5 (−8.2, 1.2)	−0.3 (−5.8, 5.3)	−3.7 (−6.6, −0.9) *	3.0 (−1.3, 7.3)	−4.9 (−9.3, −0.5)	−4.1 (−8.6, 0.4)
O_3_	−1.9 (−6.4, 2.6)	0.4 (−5.3, 6.0)	−2.1 (−4.9, 0.8)	4.2 (−0.2, 8.7)	−3.5 (−7.8, 0.8)	−2.6 (−7.4, 2.2)
O_3_	−2.9 (−8.2, 2.5)	−0.8 (−7.1, 5.4)	−2.9 (−6.1, 0.3)	4.5 (−0.3, 9.3)	−5.5 (−10.5, −0.4)	−5.3 (−10.5, −0.1)
O_3_	−4.4 (−9.7, 1.0)	−2.8 (−9.7, 4.1)	−5.8 (−8.9, −2.7) ***	2.8 (−3.0, 8.5)	−8.0 (−12.9, −3.1) **	−7.0 (−13.0, −1.0)
O_3_	−3.0 (−7.6, 1.6)	0.8 (−4.7, 6.2)	−3.9 (−6.9, −0.9) *	4.1 (−0.3, 8.4)	−5.2 (−9.8, −0.5)	−2.5 (−6.9, 2.0)

Beta estimates and 95% confidence intervals (β [95% CI]) are for pollutant 1 from models that included a second pollutant. Multipollutant models were adjusted for the age of the mother at enrollment, exposure to secondhand smoke, pre-pregnancy BMI, marital status, and education. Bold font indicates statistical significance at * *p* < 0.05, ** *p* < 0.01, *** *p* < 0.001. *p*-values were adjusted for multiple testing using the False Discovery Rate.

## Data Availability

Not applicable.
